# Tooth loss and pneumonia mortality: A cohort study of Japanese dentists

**DOI:** 10.1371/journal.pone.0195813

**Published:** 2018-04-13

**Authors:** Shino Suma, Mariko Naito, Kenji Wakai, Toru Naito, Masaaki Kojima, Osami Umemura, Makoto Yokota, Nobuhiro Hanada, Takashi Kawamura

**Affiliations:** 1 Department of Preventive Medicine, Nagoya University Graduate School of Medicine, Nagoya, Aichi, Japan; 2 Section of Preventive and Public Health Dentistry, Division of Oral Health, Growth and Development, Kyushu University Faculty of Dental Science, Fukuoka, Fukuoka, Japan; 3 Department of Maxillofacial Functional Development, Hiroshima University Graduate School of Biomedical and Health Sciences, Hiroshima, Hiroshima, Japan; 4 Section of Geriatric Dentistry, Department of General Dentistry, Fukuoka Dental College, Fukuoka, Fukuoka, Japan; 5 Aichi Dental Association, Nagoya, Aichi, Japan; 6 Yokota Dental Private School, Fukuoka, Fukuoka, Japan; 7 Department of Translational Research, School of Dental Medicine, Tsurumi University, Yokohama, Kanagawa, Japan; 8 Kyoto University Health Service, Kyoto, Kyoto, Japan; New York Medical College, UNITED STATES

## Abstract

Although associations between oral health and pneumonia have been reported in previous studies, particularly in the institutionalized elderly, few prospective studies have investigated the association between oral condition and pneumonia among community-dwelling people and whether the findings among inpatients or patients in nursing homes are applicable to the general population is still unclear. The oral bacteria propagated in the periodontal regions may drop into the lung and increase the risk of pneumonia. We, therefore, investigated the association of tooth loss with mortality from pneumonia in a cohort study of Japanese dentists. Members of the Japan Dental Association (JDA) participated in the LEMONADE (Longitudinal Evaluation of Multi-phasic, Odontological and Nutritional Associations in Dentists) Study. From 2001 to 2006, they completed a baseline questionnaire on lifestyle and health factors including the number of teeth lost (excluding third molars). We followed 19,775 participants (mean age ± standard deviation, 51.4 ± 11.7 years; 1,573 women [8.0%] and 18,202 men [92.0%]) for mortality from pneumonia (ICD-10, J12-J18). Mortality data were collected via the fraternal insurance program of the JDA. The hazard ratios (HRs) were estimated with adjustment for sex, age, body mass index, smoking status, physical activity and diabetes history. During the median follow-up period of 9.5 years, we documented 68 deaths from pneumonia. Participants who were edentulous at baseline were at significantly increased risk of mortality from pneumonia. The multivariable-adjusted HRs were 2.07 (95% confidence interval [CI], 1.09–3.95) for the edentulous and 1.60 (95% CI, 0.83–3.10) for loss of 15–27 teeth relative to loss of 0–14 teeth (trend *p* = 0.026). The HR per one tooth loss was also significant; 1.031 (95% CI, 1.004–1.060). In conclusion, a large number of teeth lost may indicate an increased risk of mortality from pneumonia in community-dwelling populations.

## Introduction

Recently, the effect of oral health on systemic disease has attracted growing attention. Many studies have reported that periodontal disease is a risk factor for cardiovascular disease [1], stroke [2], diabetes mellitus [3], preterm low birth weight [4], osteoporosis [5] and aspiration pneumonia [6].

Meanwhile, deaths from pneumonia have gradually increased since the 1980s in Japan, ranking third among causes of Japanese mortality in 2015 [7]. Because the mortality rate from pneumonia increases rapidly with increasing age [8], the recent rise in mortality from pneumonia is considered to result from aging of Japanese population. In response to the increase in pneumonia deaths in Japan, pneumococcal vaccine was added to the list of routine vaccinations in that country in October 2014 [9]. Additionally, the Japanese Respiratory Society has called for a “stop pneumonia campaign”, which emphasizes oral care by health care workers as a preventive measure for pneumonia [10], together with vaccination and improvement in nutritional status. Many groups, including academic societies, have also publicized the importance of oral care in pneumonia [11], and its significance is now more widely understood. Many facilities for the elderly currently provide oral care not only to improve swallowing and nutrition, but also to prevent pneumonia [12]. The World Health Organization has also reported that respiratory infections including pneumonia are the fourth leading cause of death in the world [13]. Pneumonia is considered an important cause of morbidity and mortality in the USA, Canada, Europe and Latin America [14].

Many intervention studies have been conducted among inpatients and patients in nursing homes investigating the relationship between oral hygiene and pneumonia [15, 16, 17]. The oral bacteria propagated in the periodontal region may drop into the lung and increase the risk of pneumonia [18, 19, 20]. However, oral disorders may have a greater influence on the risk of pneumonia among inpatients or patients in nursing homes (because of dysphagia and deteriorated immune systems) than among community-dwelling people. Nevertheless, few cohort studies have been conducted in community-dwelling individuals [21, 22], and whether the findings among inpatients or patients in nursing homes are applicable to the general population is still unclear. We therefore investigated the association of tooth loss with mortality from pneumonia in a prospective study of Japanese dentists.

## Materials and methods

### Participants and data collection

We conducted the present analysis in the LEMONADE (Longitudinal Evaluation of Multi-phasic, Odontological and Nutritional Associations in Dentists) Study. The study design and profile at baseline have been previously described [23]. In brief, the participants consisted of members of the Japan Dental Association (JDA), most of whom were practicing dentists and community-dwelling people. According to the response to the baseline questionnaire, 98.4% of all the respondents for analysis engaged in dental practice. Even among individuals aged 70 years or older, 85.6% worked as practitioners. From 2001 through 2006, they completed a self-administered baseline questionnaire on lifestyle and health factors including the number of teeth lost (excluding third molars) and medical history of diabetes. Of the 58,792 JDA members in 46 prefectures, who received questionnaires, 21,272 returned valid response (response rate, 36.2%). We excluded twelve participants with incomplete follow-up information, 1,311 with a history of cancer and/or stroke, 72 without a 1-year follow-up period and 102 with missing information about the number of teeth lost. Eventually, 19,775 dentists were included in the analysis. The mean age was 51.4 years (standard deviation, 11.7; range, 26–98), and 1,573 (8.0%) were women. The proportion of female JDA members in 2009 was 8.0%, which is almost the same as the female percentage of the present study [24]. Written informed consent was obtained from all the participants. The protocol of the LEMONADE Study was approved by the ethics committees of the Nagoya University Graduate School of Medicine and the Aichi Cancer Center (former affiliation of the principal investigator) (approval nos., 33, 632–3 and 8–21). We have conformed to the Strengthening the Reporting of Observational Studies in Epidemiology (STROBE) guidelines in the report of this study.

### Follow-up

Mortality data were collected via the fraternal insurance program of the JDA based on informed consent. Because this fraternal insurance was independent of health insurance, the mortality data of the JDA members could be traced from this program even if they changed their health insurance. Most of the JDA members do not leave the program even when they stop practicing. When a JDA member dies, his/her proxy submits a copy of the death certificate issued by a physician to claim the insurance benefit through the office of his/her prefectural dental association. We confirmed the cause of death based on the information on the certificate. Participants were followed up through to March 2014 except for those in two prefectures with an earlier finish date (December 2010 or March 2012), and those in three prefectures with a later finish date (March 2015). Resignation from a prefectural dental association or refusal of further follow-up were treated as censored cases. We defined pneumonia mortality as deaths coded as J12-J18 with the International Statistical Classification of Diseases and Related Health Problems, Tenth Revision (ICD-10). In accordance with the coding rule of ICD-10, we excluded deaths associated with underlying diseases known to lead to pneumonia morbidity from the definition.

### Statistical analysis

Body mass index (BMI) was calculated by dividing self-reported body weight (kg) by the square of self-reported height (m^2^). The smoking status of participants was classified as never, former, or current smokers. According to the self-reported number of teeth lost, the participants were classified into three groups: 0–14, 15–27, or 28 teeth. To compare background characteristics among the three groups, the chi-square test and analysis of variance were used for categorical variables and continuous variables, respectively.

The hazard ratios (HRs) among the highest two categories of tooth loss relative to the lowest were estimated with adjustment for sex, age (as a continuous variable), BMI (< 18.5, 18.5–24.9, or > 25.0 kg/m^2^) [25], smoking status (never, former, or current) [26], diabetes history (yes or no) [27] and vigorous physical activity (< 30 minutes/week or > 30 minutes/week) [28]. To examine the effect of tooth loss categories, we repeated the analysis with another grouping for tooth loss (0–9, 10–19, or > 20 teeth lost) as a sensitivity analysis. Increasing trend in the risk of mortality from pneumonia with an increasing number of teeth lost was statistically tested by assigning a score of 0, 1 or 2 to either the loss of 0–14, 15–27 or 28 teeth or the loss of 0–9, 10–19, or 20–28 teeth in the proportional hazard models. The HR per one-tooth loss was also estimated to assess overall association. All *p* values were two-sided, and *p* < 0.05 was considered statistically significant. All the analyses were performed with the Statistical Analysis System, version 9.1 (SAS Institute Inc., Cary, NC, USA).

## Results

Table 1 shows the baseline characteristics of participants according to the number of teeth lost. Participants with more lost teeth tended to be older and leaner, and more likely to have smoked and to have a history of diabetes. There was a higher proportion of women in the edentulous category.

**Table 1:** Baseline characteristics of participants by number of teeth lost.

Baseline characteristics	Number of teeth lost (excluding third molars)			*p*
	0–14 (n = 18,532)	15–27 (n = 741)	28 (n = 502)	
Age (years, mean ± SD)	50.0 ± 10.3	71.2 ± 10.2	74.6 ± 10.6	< 0.001
Women (%)	1,451(7.8)	62 (8.4)	60 (12.0)	0.003
BMI (kg/m^2^, mean ± SD)	23.7 ± 3.0	22.9 ± 3.0	22.6 ± 3.1	< 0.001
Smoking status				
Never (%)	6,872 (37.4)	189 (25.8)	136 (27.8)	< 0.001
Former (%)	6,153 (33.5)	316 (43.2)	199 (40.7)	
Current (%)	5,365 (29.2)	227 (31.0)	154 (31.5)	
History of diabetes				
Yes (%)	1,163 (6.3)	123 (16.6)	70 (13.9)	< 0.001
BMI (kg/m^2^)				
< 18.5 (%)	476 (2.6)	50 (6.9)	49 (10.0)	< 0.001
18.5–24.9 (%)	12,479 (67.9)	509 (69.9)	334 (68.3)	
25.0 ≤ (%)	5,415 (29.5)	169 (23.2)	106 (21.7)	
Vigorous physical activity				
< 30 minutes/week	13,100 (70.7)	501 (67.6)	308 (61.4)	< 0.001
> 30 minutes/week	4,543 (24.5)	67 (9.0)	42 (8.4)	
Unknown	889 (4.8)	173 (23.3)	152 (30.3)	

BMI, body mass index

During the median follow-up of 9.5 years, we documented 68 deaths from pneumonia. Participants who were edentulous at baseline were at a significantly increased risk of mortality from pneumonia (Table 2): the multivariable-adjusted HRs were 2.07 (95% confidence interval [CI], 1.09–3.95) for the edentulous and 1.60 (95% CI, 0.83–3.10) for loss of 15–27 teeth relative to loss of 0–14 teeth (trend *p* = 0.026). Comparing loss of 10–19 and > 20 teeth with loss of 0–9 teeth gave multivariable-adjusted HRs of 2.68 (95% CI, 1.31–5.51) and 2.39 (95% CI, 1.22–4.67), respectively (trend *p* = 0.015). Overall, the risk of mortality from pneumonia was associated with the number of teeth lost, as shown by the HR per tooth (HR, 1.031; 95% CI, 1.004–1.060). When we restricted the analysis to participants aged 50 years or older, the findings were not substantially altered (S1 Table, median follow-up period: 9.5 years).

**Table 2:** Hazard ratios (HRs) for mortality from pneumonia by number of teeth lost.

Number of teeth lost (excluding third molars)	n	Person-years	Death from pneumonia	Age- and sex-adjusted		Multivariable-adjusted^a^	
				HR	95% CI^b^	HR	95% CI^b^
0–14	18,532	177,988	26	1.00		1.00	
15–27	741	6,301	17	1.56	0.81–2.99	1.60	0.83–3.10
28	502	4,089	25	2.08	1.10–3.92	2.07	1.09–3.95
				Trend *p* = 0.024^c^		Trend *p* = 0.026^c^	
0–9	18,098	174,089	18	1.00		1.00	
10–19	739	6,528	15	2.61	1.28–5.32	2.68	1.31–5.51
> 20	938	7,762	35	2.38	1.23–4.60	2.39	1.22–4.67
				Trend *p* = 0.012^c^		Trend *p* = 0.015^c^	
Per tooth	19,775	188,378	68	1.031	1.005–1.059	1.031	1.004–1.060
				*p* = 0.021		*p* = 0.025	

^a^Adjusted for age (as a continuous variable), sex, smoking (never, former, or current smokers), medical history of diabetes (yes or no), body mass index (< 18.5, 18.5–24.9 or > 25.0 kg/m^2^) and vigorous physical activity (< 30 minutes/week or > 30 minutes/week). Missing covariate values were incorporated into proportional hazard models as additional categories.

^b^Confidence interval.

^c^Increasing trend in the risk of mortality from pneumonia with an increasing number of teeth lost was statistically tested by assigning a score of 0, 1 or 2 to either the loss of 0–14, 15–27 or 28 teeth or the loss of 0–9, 10–19, or 20–28 teeth in the proportional hazard models.

## Discussion

In the present study, the number of teeth lost was positively associated with the risk of pneumonia mortality in a community-dwelling population. The risk remained significant after adjustment for possible confounding factors including BMI, smoking status and diabetes.

Several underlying mechanisms have been proposed regarding associations between tooth loss and pneumonia. The possible pathway most frequently discussed is that the increase in cytokines induced by inflammation in periodontal disease may promote systemic inflammation including the lung [19, 29, 30]. Interleukin-1 and interleukin-6 levels have been assessed as inflammatory markers in patients with periodontal disease [31, 32, 33]. In the Japanese population, periodontal disease is one of the main causes of tooth extraction and accounts for 41.8% of extractions. In the middle-aged group (55–64 years), the disease accounted for nearly 60% of teeth extracted [34]. Thus, periodontal disease may explain the association of tooth loss with pneumonia risk. Many studies have reported that some bacteria involved in periodontal disease are aspirated into the lung [29]. Periodontal disease bacteria such as *Porphyromonas gingivalis* and *Actinobacillus actinomycetemcomitans* have been identified in the lungs of patients with pneumonia [32]. Additionally, periodontal disease-associated enzymes in saliva might modify the mucosal surface of the lung, and destroy its pellicles [35]. Furthermore, the reduced masticatory function caused by tooth loss may result in a limited selection of foods [36], and insufficient nutrition might increase the risk of pneumonia [37, 38, 39]. In our previous study, we found that the intake of nutrients and food groups (such as carotene, vitamins A and C, milk and dairy products, and vegetables) declined and carbohydrate intake increased as the number of teeth decreased. This finding suggested that tooth loss leads to nutritional imbalance [40]. In this study, the pathway through masticatory function and nutrition might have more strongly influenced the risk of pneumonia than the inflammation pathway because the HRs were increased in edentulous participants, who would be less affected by inflammation. Retaining teeth might be important for the prevention of pneumonia. Alternatively, tooth loss could serve as a major indicator of aging that is linked to pneumonia risk [21, 34, 41].

Our findings suggest that tooth loss is a predictor of mortality from pneumonia. Because tooth loss is the most apparent indicator of oral health for the general public, it may be useful to presume that individuals with tooth loss may be predisposed to severe pneumonia, enabling preventive action to be taken. Although the dose–response relationship was not clearly shown between the number of teeth lost and the risk of death from pneumonia, we found an overall positive association. The increase in the risk of pneumonia mortality roughly corresponds to an increase by 15.6 per 100,000 population per year in the edentulous compared to those with 0–14 teeth lost ([2.07–1] × [26/177,988] × 100,000). Although this absolute increase may not be large, it was substantial in the elderly aged 70 years or older: 209 per 100,000 per year ([1.99–1] × [17/8044] × 100,000. The HR for the edentulous compared with those with 0–14 teeth lost was 1.99 [95% CI 1.01–3.90]).

Although several investigations reported associations between indicators of periodontal disease and pneumonia [6, 42, 43], only a few examined the number of present or lost teeth in relation to pneumonia [6, 22]. In a cohort study by Awano *et al*., the risk of mortality from pneumonia was 3.9 times higher in participants with 10 or more teeth with a probing depth (PD) exceeding 4 mm [22]. However, the number of teeth was not significantly correlated with mortality from pneumonia, which is not consistent with our results. Possible reasons are that their participants were much older than ours (all participants were 80 years old at baseline) and may have retained fewer teeth. For individuals retaining a certain number of teeth, there may be an inverse correlation between number of teeth and risk of pneumonia mortality through infection by periodontal disease. This risk, however, might be decreased in the edentulous, and complete dentures could make mastication easier than several wobbly teeth affected by periodontal disease. It is therefore possible that their study did not detect the association of tooth loss with pneumonia mortality. Additionally, they included participants with a history of stroke and cancer. Pneumonia deaths associated with underlying diseases and aging might have attenuated the association of tooth loss with pneumonia mortality.

The present study has several strengths, including the prospective cohort design and adjustment for possible confounding factors. Many previous studies reported associations of oral conditions with aspiration pneumonia [6, 44]. By excluding aspiration pneumonia from endpoints in this study, we concluded that mortality from community-acquired pneumonia was also related to oral health, even in a healthy population.

However, some limitations should be considered. First, we did not establish the cause of tooth loss (e.g., periodontal disease, dental caries). Nevertheless, the number of teeth lost is the most obvious indicator of oral condition. Second, the number of teeth lost was self-reported. However, self-reported counting of teeth lost is considered to be sufficiently valid [45]. In the present study, we expected the number reported by dentists to be more accurate than in the general population. Third, we did not consider temporal changes in the number of teeth lost and covariates. Fourth, no information was available on the history of lung disease, so this could not be considered as a variable in the analysis. Finally, the participants consisted of members of a dental association and the response rate was relatively low (36.2%), which may limit the generalizability of the results. The cohort of dentists may not be representative of a community population. The meaning of losing teeth may differ between a dentist and a member of the general population; the loss of teeth in dentists would have occurred despite sufficient oral care and dental treatment. However, the underlying biological mechanisms would be common among human populations, and the population in the current study was homogeneous in terms of socioeconomic status, which may reduce confounding by socioeconomic factors. Although the healthy worker effect should be taken into account [46], cohort studies of health care workers have provided important health information, as in the Nurses’ Health Study and the Health Professionals Follow-Up Study [47, 48].

In conclusion, a high number of teeth lost may indicate an increased risk of mortality from pneumonia in community-dwelling populations.

## Supporting information

S1 Table Hazard ratios (HRs) for mortality from pneumonia by number of teeth lost among participants aged 50 years or older. ^a^Adjusted for age (as a continuous variable), sex, smoking (never, former, or current smokers), medical history of diabetes (yes or no), body mass index (< 18.5, 18.5–24.9 or > 25.0 kg/m^2^), and vigorous physical activity (< 30 minutes/week or ≥ 30 minutes/week). Missing covariate values were incorporated into proportional hazard models as additional categories.
^b^Confidence interval.
^c^Increasing trend in the risk of mortality from pneumonia with an increasing number of teeth lost was statistically tested by assigning a score of 0, 1 or 2 to either the loss of 0–14, 15–27 or 28 teeth or the loss of 0–9, 10–19, or 20–28 teeth in the proportional hazard models.
(DOCX)
