# Supplementary material for: Tooth loss and pneumonia mortality: A cohort study of Japanese dentists
Source: PLoS One. 2018 Apr 13;13(4):e0195813. doi: 10.1371/journal.pone.0195813 (PMC5898744; doi:10.1371/journal.pone.0195813)
Supplement: S1 Table — aAdjusted for age (as a continuous variable), sex, smoking (never, former, or current smokers), medical history of diabetes (yes or no), body mass index (< 18.5, 18.5–24.9 or > 25.0 kg/m2), and vigorous physical activity (< 30 minutes/week or ≥ 30 minutes/week). Missing covariate values were incorporated into proportional hazard models as additional categories. bConfidence interval. cIncreasing trend in the risk of mortality from pneumonia with an increasing number of teeth lost was statistically tested by assigning a score of 0, 1 or 2 to either the loss of 0–14, 15–27 or 28 teeth or the loss of 0–9, 10–19, or 20–28 teeth in the proportional hazard models. (DOCX) [file pone.0195813.s001.docx]

**S1 Table. Hazard ratios (HRs) for mortality from pneumonia by number of teeth lost among participants aged 50 years or older**

| Number of teeth lost (excluding third molars) | n | Person-years | Death from pneumonia | Age- and sex-adjusted | |  | Multivariable-adjusted^a^ | |
| --- | --- | --- | --- | --- | --- | --- | --- | --- |
|  |  |  |  | HR | 95% CI^b^ |  | HR | 95% CI^b^ |
| 0–14 | 8,785 | 83,291 | 25 | 1.00 |  |  | 1.00 |  |
| 15–27 | 718 | 6,075 | 17 | 1.54 | 0.80–2.95 |  | 1.57 | 0.81–3.04 |
| 28 | 486 | 3,930 | 25 | 2.04 | 1.08–3.86 |  | 2.01 | 1.05–3.84 |
|  |  |  |  | Trend *p* = 0.027^c^ | |  | Trend *p* = 0.034^c^ | |
|  |  |  |  |  |  |  |  |  |
| 0–9 | 8,358 | 79,457 | 17 | 1.00 |  |  | 1.00 |  |
| 10–19 | 727 | 6,417 | 15 | 2.60 | 1.27–5.30 |  | 2.63 | 1.28–5.42 |
| > 20 | 904 | 7,422 | 35 | 2.34 | 1.21–4.52 |  | 2.32 | 1.18–4.53 |
|  |  |  |  | Trend *p* = 0.014^c^ | |  | Trend *p* = 0.019^c^ | |
|  |  |  |  |  |  |  |  |  |
| Per tooth | 9,989 | 93,295 | 67 | 1.031 | 1.004–1.059 |  | 1.030 | 1.003–1.059 |
|  |  |  |  | *p* = 0.024 | |  | *p* = 0.032 | |

^a^Adjusted for age (as a continuous variable), sex, smoking (never, former, or current smokers), medical history of diabetes (yes or no), body mass index (< 18.5, 18.5–24.9 or > 25.0 kg/m^2^) and vigorous physical activity (< 30 minutes/week or > 30 minutes/week). Missing covariate values were incorporated into proportional hazard models as additional categories.

^b^Confidence interval.

^c^Increasing trend in the risk of mortality from pneumonia with an increasing number of teeth lost was statistically tested by assigning a score of 0, 1 or 2 to either the loss of 0–14, 15–27 or 28 teeth or the loss of 0–9, 10–19, or 20–28 teeth in the proportional hazard models.
